# Deep Learning for Brain MRI Segmentation: State of the Art and Future Directions

**DOI:** 10.1007/s10278-017-9983-4

**Published:** 2017-06-02

**Authors:** Zeynettin Akkus, Alfiia Galimzianova, Assaf Hoogi, Daniel L. Rubin, Bradley J. Erickson

**Affiliations:** 1 Radiology Informatics Lab, Mayo Clinic, 200 First Street SW, Rochester, MN 55905 USA; 2 Department of Radiology, Stanford University School of Medicine, Stanford, CA USA

**Keywords:** Deep learning, Quantitative brain MRI, Convolutional neural network, Brain lesion segmentation

## Abstract

Quantitative analysis of brain MRI is routine for many neurological diseases and conditions and relies on accurate segmentation of structures of interest. Deep learning-based segmentation approaches for brain MRI are gaining interest due to their self-learning and generalization ability over large amounts of data. As the deep learning architectures are becoming more mature, they gradually outperform previous state-of-the-art classical machine learning algorithms. This review aims to provide an overview of current deep learning-based segmentation approaches for quantitative brain MRI. First we review the current deep learning architectures used for segmentation of anatomical brain structures and brain lesions. Next, the performance, speed, and properties of deep learning approaches are summarized and discussed. Finally, we provide a critical assessment of the current state and identify likely future developments and trends.

## Background

Magnetic resonance imaging (MRI) is usually the modality of choice for structural brain analysis, since it provides images with high contrast for soft tissues and high spatial resolution and presents no known health risks. While modalities such as computed tomography (CT) and positron emission tomography (PET) are also used to study the brain, MRI is the most popular, and we will focus on MRI in this work. Quantitative analysis of brain MRI has been used extensively for characterization of brain disorders such as Alzheimer’s disease, epilepsy, schizophrenia, multiple sclerosis (MS), cancer, and infectious and degenerative diseases. For example, tissue atrophy is one of the common biomarkers used in diagnosis and therapy assessment in Alzheimer’s disease, epilepsy, schizophrenia, MS, and many other neurological diseases and disorders. To quantify tissue atrophy, segmentation and corresponding measurements of brain tissues are needed. Similarly, quantification of change in brain structures requires segmentation of the MRI obtained at different time points. In addition, detection and precise localization of the abnormal tissue and surrounding healthy structures are crucial for diagnosis, surgical planning, postoperative analysis, and chemo/radiotherapy planning. Quantitative and qualitative characterization of normal and pathological structures, both in space and time, are often part of clinical trials, in which the effects of treatment are studied on a cohort of patients and normal controls.

Quantitative analysis of brain MR images is routine for many neurological diseases and conditions. Segmentation, i.e., labeling of pixels in 2D (voxels in 3D), is a critical component of quantitative analysis. Manual segmentation is the gold standard for in vivo images. However, this requires outlining structures slice-by-slice, and is not only expensive and tedious, but also inaccurate due to human error. Therefore, there is a need for automated segmentation methods to provide accuracy close to that of expert raters’ with a high consistency.

As 3D and 4D imaging are becoming routine, and with physiological and functional imaging increasing, medical imaging data is increasing in size and complexity. Therefore, it is essential to develop tools that can assist in extracting information from these large datasets. Machine learning is a set of algorithmic techniques that allow computer systems to make data-driven predictions from large data. These techniques have a variety of applications that can be tailored to the medical field.

There has been a significant effort in developing classical machine learning algorithms for segmentation of normal (e.g., white matter and gray matter) and abnormal brain tissues (e.g., brain tumors) in MRI. However, creation of the imaging features that enable such segmentation requires careful engineering and specific expertise. Furthermore, traditional machine learning algorithms do not generalize well. Despite a significant effort from the medical imaging research community, automated segmentation of the brain structures and detection of the abnormalities remain an unsolved problem due to normal anatomical variations in brain morphology, variations in acquisition settings and MRI scanners, image acquisition imperfections, and variations in the appearance of pathology.

An emerging machine learning technique referred to as deep learning [1], can help avoid limitations of classical machine learning algorithms, and its self-learning of features may enable identification of new useful imaging features for quantitative analysis of brain MRI. Deep learning techniques are gaining popularity in many areas of medical image analysis [2], such as computer-aided detection of breast lesions [3], computer-aided diagnosis of breast lesions and pulmonary nodules [4], and in histopathological diagnosis [5]. In this survey, we provide an overview of state-of-the-art deep learning techniques in the field of brain MR segmentation and discuss remaining gaps that have a potential to be fulfilled by the use of deep learning techniques.

### Deep Learning

Deep learning refers to neural networks with many layers (usually more than five) that extract a hierarchy of features from raw input images. It is a new and popular type of machine learning techniques that extract a complex hierarchy of features from images due to their self-learning ability as opposed to the hand-crafted feature extraction in classical machine learning algorithms. They achieve impressive results and generalizability by training on large amount of data. The rapid increase in GPU processing power has enabled the development of state-of-the-art deep learning algorithms. This allowed training of deep learning algorithms with millions of images and provided robustness to variations in images.

There are several types of deep learning approaches that have been developed for different purposes, such as object detection and segmentation in images, speech recognition, and genotype/phenotype detection and classification of diseases. Some of the known deep learning algorithms are stacked auto-encoders, deep Boltzmann machines, deep neural networks, and convolutional neural networks (CNNs). CNNs are the most commonly applied to image segmentation and classification.

CNNs were first introduced in 1989 [6], but gained great interest after deep CNNs achieved spectacular results in ImageNet [7, 8] competition in 2012 [9]. Applied on a dataset of about a million images that included 1000 different classes, CNNs nearly halved the error rates of the previously best computing approaches [9].

**Fig. 1:**
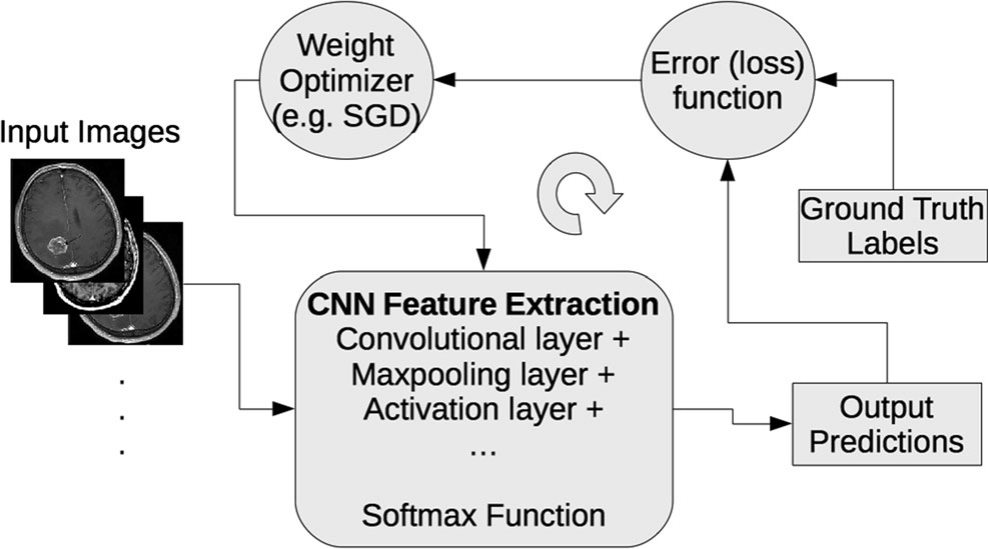
A schematic representation of a convolutional neural network (CNN) training process

CNN architectures are increasingly complex, with some systems having more than 100 layers, which means millions of weights and billions of connections between neurons. A typical CNN architecture contains subsequent layers of convolution, pooling, activation, and classification (fully connected). Convolutional layer produces feature maps by convolving a kernel across the input image. Pooling layer is used to downsample the output of preceding convolutional layers by using the maximum or average of the defined neighborhood as the value passed to the next layer. Rectified Linear Unit (ReLU) and its modifications such as Leaky ReLU are among the most commonly used activation functions. ReLU nonlinearly transforms data by clipping any negative input values to zero while positive input values are passed as output [10]. To perform a prediction of an input data, the output scores of the final CNN layer are connected to loss function (e.g., cross-entropy loss that normalizes scores into multinomial distribution over labels). Finally, parameters of the network are found by minimizing a loss function between prediction and ground truth labels with regularization constraints, and the network weights are updated at each iteration (e.g., using stochastic gradient descent – SGD) using backpropagation until convergence (see Fig. 1).

## Review

We performed a thorough analysis of the literature using the Google Scholar and NLM Pubmed search engines. We included all found peer reviewed journal publications and conference proceedings that describe applying deep learning to brain MRI segmentation. Since a large fraction of deep learning works are submitted to Arxiv (http://arxiv.org) first, we also included relevant Arxiv preprints. Conference proceedings that had a follow-up journal publication were included only in their final publication form. We divided papers into two groups: works on normal structures and on brain lesions. In both groups, different deep learning architectures have been introduced to address domain-specific challenges. We further subdivided them based on their architecture style such as patch-wise, semantic-wise, or cascaded architectures. In the following subsections, we present evaluation and validation methods, preprocessing methods used in current deep learning approaches, current deep learning architecture styles, and performance of deep learning algorithms for quantification of brain structures and lesions.

### Training, Validation and Evaluation

In the machine learning field, data are divided into training, validation, and test sets for learning from examples, establishing the soundness of learning results, and evaluating the generalization ability of a developed algorithm on unseen data, respectively. When there are limited data, cross validation methods (e.g., one-leave out, fivefold, or tenfold validations) are preferred. In a k-fold cross-validation, the data are randomly partitioned into k equal sized parts. One of the k parts is retained as the validation data for testing the algorithm, and the remaining k – 1 parts are used as training data. Training is typically done with a supervised approach which requires ground truth for the task. Ground truth is usually obtained with manual delineations of brain lesions or structures by experts for segmentation tasks. Even though this is the gold standard for the learning and evaluation, it is a tedious and laborious task and contains subjectivity. In their work, Mazzara et al. [11] reported intra-expert variabilities of 20 ± 15% and inter-experts variabilities of 28 ± 12% for manual segmentations of brain tumor images. To alleviate this variability, multiple expert segmentations are combined in an optimal way by using label fusion algorithms such as STAPLE [12, 13]. For classification tasks of brain lesions, the ground truth is obtained with biopsy and pathological tests.

To evaluate performance of a newly developed deep learning approach on a task, it is essential to compare its performance against available state of the art methods. In general, most of the algorithms are evaluated on different sets of data and reported different similarity metrics. This makes it hard to compare the performance of different algorithms against each other. Over the last decade, the brain imaging community has become more aware of this and created publicly available datasets with ground truth for evaluating the performance of algorithms against each other in an unbiased way. One of the first such datasets was released in the framework of an MS lesion segmentation challenge, which was held in conjunction with MICCAI 2008. The dataset is maintained as an online challenge dataset (https://www.nitrc.org/projects/msseg), meaning the training data is released with the ground truth to the public, while the test dataset is released without the ground truth and thus can be evaluated only by the organizers. The latter helps avoid overfitting of the methods and makes comparison more objective. Following the same paradigm, many other datasets have been released since then. Some of the other well-known publicly available datasets for brain MRI are Brain Tumor Segmentation (BRATS), Ischemic Stroke Lesion Segmentation (ISLES), Mild Traumatic Brain Injury Outcome Prediction (mTOP), Multiple Sclerosis Segmentation (MSSEG), Neonatal Brain Segmentation (NeoBrainS12), and MR Brain Image Segmentation (MRBrainS).

#### Brats

This brain tumor image segmentation challenge in conjunction with the MICCAI conference has been held annually since 2012 in order to evaluate the current state-of-the-art in automated brain tumor segmentation and compare between different methods. For this purpose, a large dataset of brain tumor MR scans and ground truth (five labels: healthy brain tissue, necrosis, edema, non-enhanced, and enhanced regions of tumors) are made publicly available. The training data has increased over the years. Currently (Brats 2015–2016), the training set comprises 220 subjects with high grade and 54 subjects with low-grade, and the test set comprises 53 subjects with mixed grades. All datasets have been aligned to the same anatomical template and interpolated to 1 mm^3^ voxel resolution. Each dataset has pre-contrast T1, post contrast T1, T2, and T2 FLAIR MRI volumes. The co-registered, skull-stripped, and annotated training dataset and evaluation results of algorithms are available via the Virtual Skeleton Database (https://www.virtualskeleton.ch/).

#### Isles

This challenge is organized to evaluate stroke lesion/clinical outcome prediction from acute MRI scans. Acute MRI scans of a large number of acute stroke cases and associated clinical parameters are provided. The associated ground truth is the final lesion volume (Task I) as manually segmented in 3 to 9-month follow-up scans, and the clinical mRM score (Task II) denoting the degree of disability. For ISLES 2016, 35 training and 40 testing cases made publicly available via SMIR platform (https://www.smir.ch/ISLES/Start2016). The performance of the winner algorithm on this dataset for subacute ischemic stroke lesion segmentation currently is 0.59 ± 0.31 (Dice similarity coefficient, DSC) and 37.88 ± 30.06 (Hausdorff Distance, HD).

#### mTOP

This challenge calls for methods that focus on finding differences between healthy subjects and Traumatic Brain Injury (TBI) patients and sort the given data in distinct categories in an unsupervised manner. Publicly available MRI data can be downloaded from https://tbichallenge.wordpress.com/data.

#### MSSEG

The goals of this challenge are evaluating state-of-the-art and advanced segmentation methods from the participants on MS data. For this, they evaluate both lesion detection (how many lesions are detected) and lesion segmentation (how precisely the lesions are delineated) on a multicenter database (38 patients from four different centers, imaged on 1.5 or 3T scanners, each patient being manually annotated by seven experts). In addition to this classical evaluation, they provide a common infrastructure to evaluate the algorithms such as running time comparison and the degree of automation. The data can be obtained from https://portal.fli-iam.irisa.fr/msseg-challenge/data.

#### NeoBrainS12

The aim of the NeoBrainS12 challenge is to compare algorithms for segmentation of neonatal brain tissues and measurement of corresponding volumes using T1 and T2 MRI scans of the brain. The comparison is performed for the following structures: cortical and central gray matter, non-myelinated and myelinated white matter, brainstem and cerebellum, and cerebrospinal fluid in the ventricles and in the extracerebral space. Training set includes T1 and T2 MR images of two infants at 30 and 40 weeks ages. Test set includes T1 and T2 MRI of five infants. The data and evaluation results of algorithms that has been submitted to the challenge can be downloaded from http://neobrains12.isi.uu.nl/.

#### MRBrainS

The aim of the MRBrainS evaluation framework is to compare algorithms for segmentation of gray matter, white matter, and cerebrospinal fluid on multi-sequence (T1-weighted, T1-weighted-inversion recovery, and FLAIR) 3 Tesla MRI scans of the brain. Five brain MRI scans with manual segmentations are provided for training and 15 only MRI scans are provided for testing. The data can be downloaded from http://mrbrains13.isi.uu.nl. The performance (DSC) of the current winner algorithm on this dataset is 86.15% for gray matter, 89.46% for white matter, and 84.25% for cerebrospinal fluid segmentation.

The most common quantitative measures used for evaluation brain MRI segmentation methods are listed below and shown in Table 1. Typically, the methods for normal structure or tumor segmentation include voxel-wise metrics, such as DSC, true positive rate (TPR), positive predictive value (PPV), and lesion surface metrics, such as HD and average symmetric surface distance (ASSD). On the other hand, methods for multifocal brain lesions often also include lesion-wise metrics, such as lesion-wise true positive rate (LTPR) and lesion-wise positive predictive value (LPPV). Measures such as accuracy and specificity tend to be avoided in the lesion segmentation context since these measures do not discriminate between different segmentation outputs when the object (lesion) is considerably smaller than the background (normal-appearing brain tissue). In addition, measures of clinical relevance are also commonly incorporated. These include such measures as correlation analysis of total lesion load or count as detected by automated and manual segmentation and volume or volume change correlation. Significance tests commonly accompany contributions that build on or compare to other methods, most often nonparametric tests such as Wilcoxon’s signed rank of Wilcoxon’s rank sum tests are preferred.

**Table 1:** A summary of popular quantitative measures of brain MRI segmentation quality and their mathematical formulation with respect to the number of false positives (FP), true positives (TP), and false negatives (FN) at voxel level and lesion level (FPL, TPL, and FNL, respectively). ∂S and ∂R are the sets of lesion border pixels/voxels for the tested and the reference segmentations, and d_m_(v, V) is the minimum of the Euclidean distances between a voxel v and voxels in a set V.

Metric of segmentation quality	Mathematical description
True positive rate, TPR	$\mathrm{TPR}=\frac{\mathrm{TP}}{\mathrm{TP}+\mathrm{FN}}$
Positive predictive rate, PPV	$\mathrm{PPV}=\frac{\mathrm{TP}}{\mathrm{TP}+\mathrm{FP}}$
Dice similarity coefficient, DSC	$\mathrm{DSC}=\frac{2\mathrm{TP}}{2\mathrm{TP}+\mathrm{FP}+\mathrm{FN}}$
Volume difference rate, VDR	$\mathrm{VDR}=\frac{\mid \mathrm{FP}-\mathrm{FN}\mid }{\mathrm{TP}+\mathrm{FN}}$
Hausdorff distance	HD = max {sup_r ∈ ∂R_d_m_(s, r), sup_s ∈ ∂S_d_m_(r, s)}
Average symmetric surface distance, ASSD	$\mathrm{S}\mathrm{D}=\frac{\sum_{\mathrm{s}\in \partial \mathrm{S}}{\mathrm{d}}_{\mathrm{m}}\left(\mathrm{s},\partial \mathrm{R}\right)+{\sum}_{\mathrm{s}\in \partial \mathrm{R}}{\mathrm{d}}_{\mathrm{m}}\left(\mathrm{r},\partial \mathrm{S}\right)}{\mid \partial \mathrm{S}\mid +\mid \partial \mathrm{R}\mid }$
Lesion-wise true positive rate, LTPR	$\mathrm{LTPR}=\frac{\mathrm{TPL}}{\mathrm{TPL}+\mathrm{FNL}}$
Lesion-wise positive predictive value, LPPV	$\mathrm{LPPV}=\frac{\mathrm{TPL}}{\mathrm{TPL}+\mathrm{FPL}}$

### Image Preprocessing

Automated analysis of MR images is challenging due to intensity inhomogeneity, variability of the intensity ranges and contrast, and noise. Therefore, prior to automated analysis, certain steps are required to make the images appear more similar, and these steps are commonly referred to as preprocessing. Typical preprocessing steps for structural brain MRI include the following key steps.

#### Registration

Registration is spatial alignment of the images to a common anatomical space [14]. Interpatient image registration aids in standardizing the MR images onto a standard stereotaxic space, commonly MNI or ICBM. Intrapatient registration aims to align the images of different sequences, e.g., T1 and T2, to obtain a multi-channel representation for each location within the brain.

#### Skull Stripping

Skull stripping is the process of removing the skull from images to focus on intracranial tissues. The most common methods used for this purpose have been BET [15], Robex [16], and SPM [16, 17].

#### Bias Field Correction

Bias Field Correciton is the correction of the image contrast variations due to magnetic field inhomogeneity [18]. The most commonly adopted approach is N4 bias field correction.

#### Intensity Normalization

Intensity Normalization is the process of mapping intensities of all images into a standard or reference scale, e.g., between 0 and 4095. The algorithm by Nyul et al. [19], which uses piecewise linear mapping of image intensities into a reference scale, is one of the most popular normalization techniques. In the context of deep learning frameworks, computing z-scores, where one subtracts the mean image intensity from all pixels in an image and divides pixels by the standard deviation of intensities, is another popular normalization technique.

#### Noise Reduction

Noise reduction is the reduction of the locally-variant Rician noise observed in MR images [20].

With advent of deep learning techniques, some of the preprocessing steps became less critical for the final segmentation performance. For instance, bias correction and quantile-based intensity normalization are often successfully replaced by the z-score computation alone [2, 21]; however, another work shows improvement when applying normalization prior to deep learning based segmentation procedure [22]. At the same time, the new methods for these preprocessing routines are also arising, including deep learning based registration [23], skull stripping [24], and noise reduction [25].

### Current CNN Architecture Styles

#### Patch-Wise CNN Architecture

**Fig. 2:**
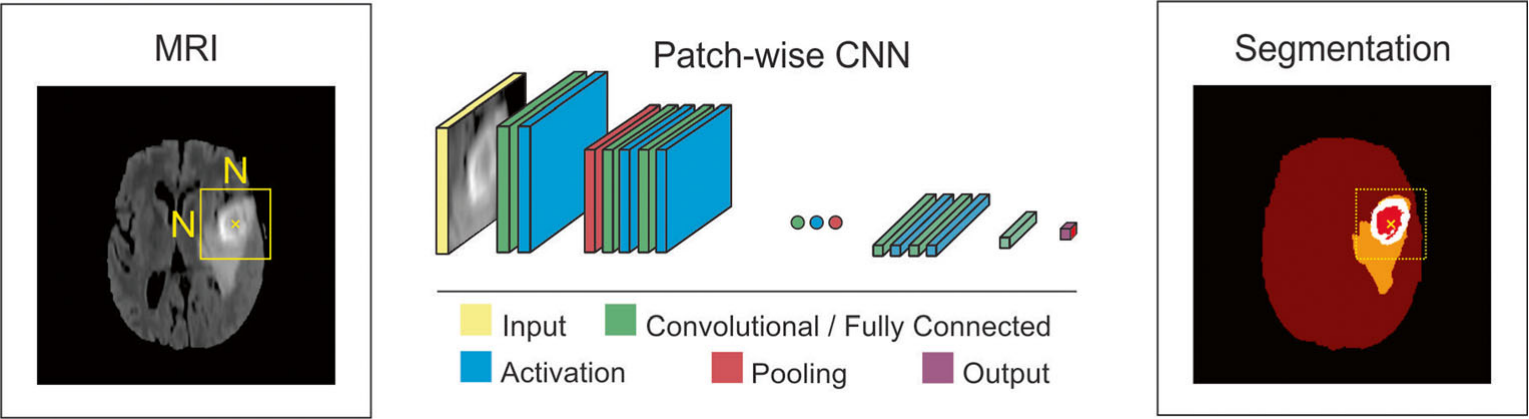
Schematic illustration of a patch-wise CNN architecture for brain tumor segmentation task

**Fig. 3:**
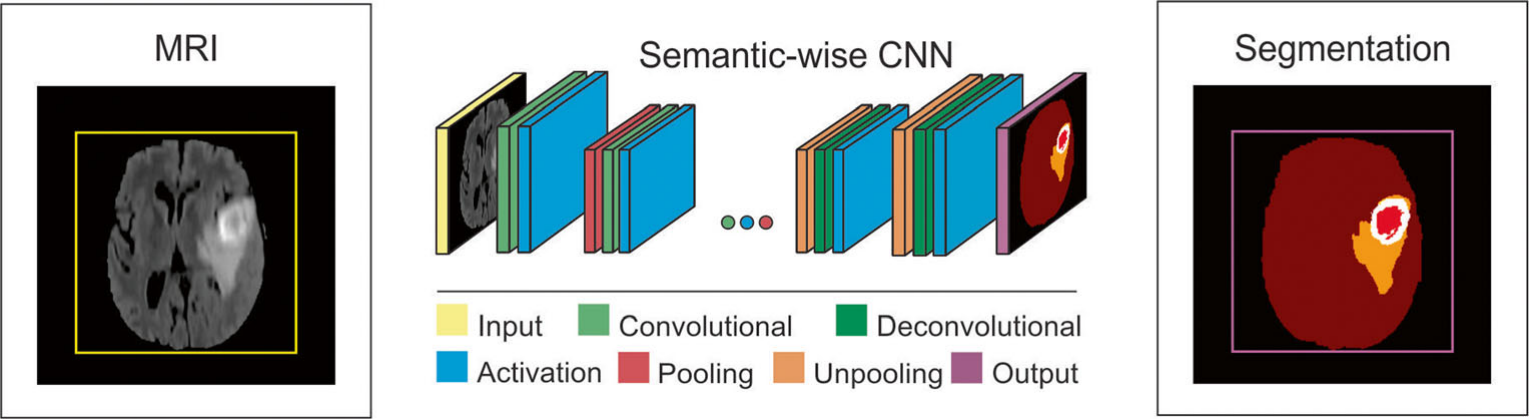
Schematic illustration of a semantic-wise CNN architecture for brain tumor segmentation task

**Fig. 4:**
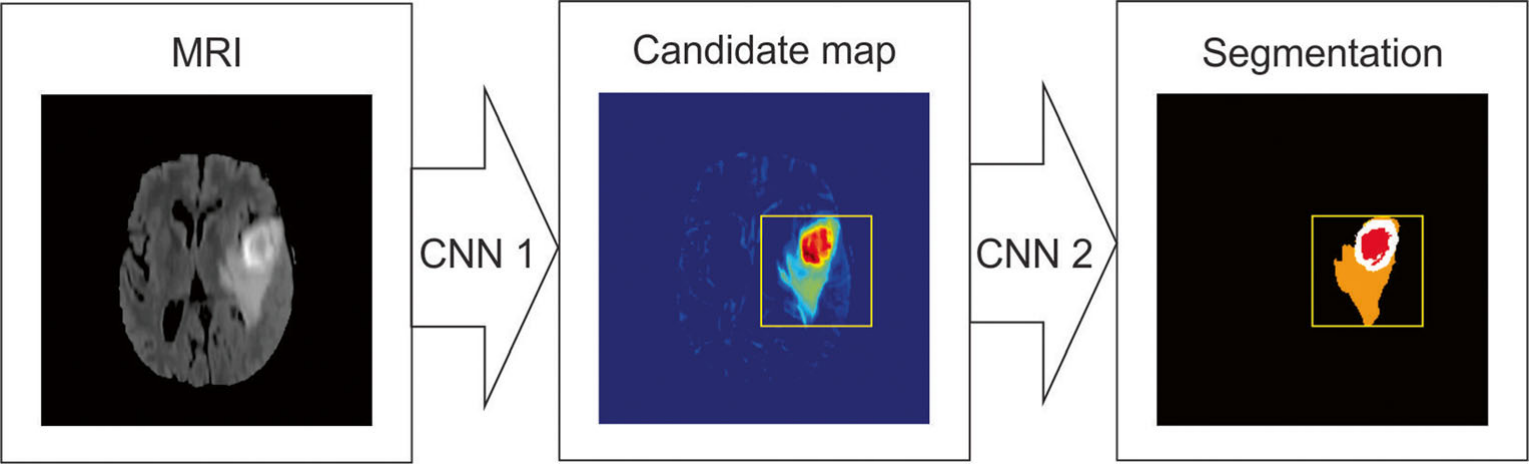
Schematic illustration of a cascaded CNN architecture for brain tumor segmentation task, where the output of the first network (CNN 1) is used in addition to image data for a refined input to the second network (CNN 2), which provides final segmentation

This is a simple approach to train a CNN algorithm for segmentation. An NxN patch around each pixel is extracted from a given image, and the model is trained on these patches and given class labels to correctly identify classes such as normal brain and tumor. The designed networks contain multiple convolutional, activation, pooling, and fully connected layers sequentially. Most of the current popular architectures [21, 22, 26, 27] use this approach. To improve the performance of patch-wise architectures, multiscale CNNs [28, 29] use multiple pathways, where each uses a patch of different size around the same pixel. The output of these pathways are combined by a neural network and the model trained to correctly identify the given class labels (Figs. 2, 3, and 4).

#### Semantic-Wise CNN Architecture

This type of architecture makes predictions for each pixel of the whole input image like semantic segmentation [30, 31]. Similar to autoencoders, they include encoder part that extracts features and decoder part that upsamples or deconvolves the higher level features from the encoder part and combines lower level features from the encoder part to classify pixels. The input image is mapped to the segmentation labels in a way that minimizes a loss function.

#### Cascaded CNN Architecture

This type of architecture combines two CNN architectures [32]. The output of the first CNN is used as an input to the second CNN to obtain classification results. The first CNN is used to train the model with initial prediction of class labels while second CNN is used to further tune the results of the first CNN.

### Segmentation of Normal Brain Structure

Accurate automated segmentation of brain structures, e.g., white matter (WM), gray matter (GM), and cerebrospinal fluid (CSF), in MRI is important for studying early brain developments in infants and quantitative assessment of the brain tissue and intracranial volume in large scale studies. Atlas-based approaches [33–36], which match intensity information between an atlas and target images and pattern recognition approaches [37–39], which classify tissues based on a set of local intensity features, are the classical approaches that have been used for brain tissue segmentation. In recent years, CNNs have been adopted for segmentation of brain tissues, which avoid the explicit definition of spatial and intensity features and provide better performance than classical approaches, as we describe next (see Table 2 for the list of studies).

**Table 2:** Deep learning approaches for brain structure segmentation

Authors	CNN Style	Dim	Accuracy	Data
Zhang et al. 2015 [27]	Patch-wise	2D	DSC 83.5% (CSF), 85.2% (GM), 86.4% (WM)	Private data (10 healthy infants)
Nie et al. 2016 [30]	Semantic-wise	2D	DSC 85.5% (CSF), 87.3% (GM), 88.7% (WM)	Private data (10 healthy infants)
de Brebisson et al. 2015 [40]	Patch-wise	2D/3D	Overall DSC 72.5% ∓ 16.3%	MICCAI 2012-multi-atlas labeling
Moeskops et al. 2016 [28]	Patch-wise	2D/3D	Overall DSC 73.53%	MICCAI 2012-multi-atlas labeling
Bao et al. 2016 [41]	Patch-wise	2D	DSC 82.2%/85%	IBSR/LPBA40

Zhang et al. [27] presented a 2D (input patch size 13 × 13 pixels) patch-wise CNN approach to segment WM, GM, and CSF from multimodal (i.e., T1, T2, and fractional anisotropy) MR images of infants. They showed that their CNN approach outperforms prior methods and classical machine learning algorithms using support vector machine (SVM) and random forest (RF) classifiers (overall DSC performance 85.03% ∓ 2.27% (CNN) vs. 76.95% ∓ 3.55% (SVM), 83.15% ∓ 2.52% (RF)). Nie et al. [30] presented a semantic-wise fully convolutional networks (FCNs) to segment infant brain images from the same dataset that Zhang et al. [27] used in their study. They obtained improved results compared to [27]. Their overall DSC were 85.5% (CSF), 87.3% (GM), and 88.7% (WM) vs. 83.5% (CSF), 85.2 (GM), and 86.4 (WM) by [27]. De Brebisson et al. [40] presented a 2D (***I*** = 29^2^) and 3D (***I*** = 13^3^) patch-wise CNN approach to segment human brain to anatomical regions. They achieved competitive results (DSC = 72.5% ∓ 16.3%) in MICCAI 2012 challenge on multi-atlas labeling as the first CNN approach applied to the task. Moeskops et al. [28] presented a multi-scale (25^2^,51^2^,75^2^ pixels) patch-wise CNN approach to segment brain images of infants and young adults. They obtained overall DSC = 73.53% vs. 72.5% by [40] in MICCAI challenge on multi-atlas labeling. Bao et al. [41] also presented a multi-scale patch-wise CNN together with dynamic random walker with decay region of interest to obtain smooth segmentation of subcortical structures in IBSR (developed by the Centre for Morphometric Analysis at Massachusetts General Hospital-available at https://www.nitrc.org/projects/ibsr to download) and LPBA40 [42] datasets. They reported overall DSC of 82.2 and 85% for IBSR and LPBA40, respectively. CNN-based deep learning approaches have shown the top performances on NeoBrainS12 and MRBrainS (see Table 3) challenges. Their computation time at testing phase was also much less than classical machine learning algorithms.

**Table 3:** Top ten ranking of algorithms in MRBrainS challenge (Complete list is available at: http://mrbrains13.isi.uu.nl/results.php)

Rank	Team name	Submission name	Sequence used	Speed
1	CU_DL	3D Deep learning; voxnet1	T1; T1_IR; FLAIR	~2 min
2	CU_DL2	3D Deep learning; voxnet2	T1; T1_IR; FLAIR	~2 min
3	MDGRU	Multi-dimensional gated Recurrent units	T1; T1_IR; FLAIR	~2 min
4	PyraMiD-LSTM2	NOCC with rounds	T1; T1-IR; FLAIR	~2 min
5	FBI/LMB Freiburg	U-Net (3D)	T1; T1-IR; F	~2 min
6	IDSIA	PyraMiD-LSTM	T1; T1_IR; FLAIR	~2 min
7	STH	Hybrid ANN-based Auto-context method	T1; T1-IR; FLAIR	~5 min
8	ISI-Neonatology	Multi-stage voxel classification	T1	~1.5 h
9	UNC-IDEA	LINKS: Learning-based multi-source integration	T1; T1_IR; FLAIR	~3 min
10	MNAB2	Random forests	T1; T1_IR; FLAIR	~25 min

### Segmentation of Brain Lesions

Quantitative analysis of brain lesions include measurement of established imaging biomarkers such as the largest diameter, volume, count, and progression, to quantify treatment response of the associated diseases, such as brain cancer, MS, and stroke. Reliable extraction of these biomarkers depends on prior accurate segmentation. Despite the significant effort in brain lesion segmentation and advanced imaging techniques, accurate segmentation of brain lesions remains a challenge. Many automated methods have been proposed for lesion segmentation problem, including unsupervised modeling methods that aim to automatically adapt to new image data [43–45] supervised machine learning methods that, given a representative dataset, learn the textural and appearance properties of lesions [46], and atlas-based methods that combine both supervised and unsupervised learning into a unified pipeline by registering labeled data or a known cohort data into a common anatomical space [47–49]. Several review papers provide overview of classical methods for brain tumor segmentation [50], and MS lesion segmentation [51, 52]. For more information and detail on the classical approaches, we refer the reader to those studies.

Several deep learning studies have shown superior performances to the classical state-of-art methods (see Table 4). Havaei et al. [26] presented a 2D (33 × 33 pixels) patch-wise architecture using local and global CNN pathways, which exploits local and global contextual features around a pixel to segment brain tumors. The local pathway includes two convolutional layers with kernel sizes of 7 × 7 and 5 × 5, respectively, while the global pathway includes one convolutional layer with kernel size of 11 × 11. To tackle the difficulties raised by imbalance of tumor vs. normal brain labels, where the fraction of latter is above 90% of total samples, they introduced two phase training which included training first with data that had equal class probability and then training only the output layer with the unbalanced data (i.e., keeping the weights of all the other layers unchanged). They also explored cascaded architectures in their study. They reported that their CNN approach outperformed and was much faster at testing phase (3 vs. 100 min) than the winner of BRATS 2013 competition.

**Table 4:** Deep learning approaches for quantification of brain lesions

Authors	Aim	CNN Style	Dim	Accuracy	Dataset
Havaei et al. 2016 [26]	Tumor segmentation	Patch-wise	2D	DSC 0.88 (complete), 0.79 (core), 0.73 (enhancing)	BRATS-2013
S. Pereira et al. 2016 [22]	Tumor segmentation	Patch-wise	2D	DSC 0.88 (complete), 0.83 (core), 0.77 (enhancing)	BRATS-2013
Zhao and Jia 2015 [53]	Tumor segmentation	Patch-wise	2D	Overall accuracy 0.81	BRATS-2013
Kamnitsas et al. 2016 [21]	Tumor segmentation	Patch-wise	3D	DSC 0.9 (complete), 0.75 (core), 0.73 (enhancing)	BRATS-2015
Dvorak et al. 2015 [54]	Tumor segmentation	Patch-wise	2D	DSC 0.83 (complete), 0.75 (core), 0.77 (enhancing)	BRATS-2014
Brosch et al. 2016 [31]	MS segmentation	Semantic-wise	3D	DSC 0.68 (ISBI); DSC 0.84 (MICCAI)	MICCAI 2008-ISBI 2015
Dou et al. 2016 [32]	Cerebral microbleed detection	Cascaded (semantic/patch-wise)	3D	Sensitivity 98.29%	Private data (320 subjects)
Maier et al. 2015 [55]	Ischemic stroke detection	Patch-wise	2D	DSC 0.67 ± 0.18; HD 29.64 ± 24.6	Private data (37 subjects)
Akkus et al. 2016 [29]	Tumor genomic prediction	Patch-wise	2D	0.93 (sensitivity), 0.82 (specificity), and 0.88 (accuracy)	Private data (159 subjects)

In another study, Havaei et al. [56] presented an overview of brain tumor segmentation with deep learning, which also described the use of cascaded architecture. Pereira et al. [22] presented a 2D patch-wise architecture, but compared to Havaei et al., they used small 3 × 3 convolutional kernels which allowed deeper architectures, patch intensity normalization, and data augmentation by rotation of patches. They also designed two separate models for each grade—high-grade (HG) and low-grade (LG) tumors. The model for HG tumors included six convolutional layers and three fully connected layers while the model for LG included four convolutional layers and three fully connected layers. They also used leaky ReLU for activation function, which allowed gradient flow in contrast to rectified linear units that impose constant zero to negative values. Their method showed the best performance on the Brats 2013 data – DSC values of 0.88, 083, 0.77 for complete, core, and enhancing regions, respectively. They were also ranked as second place in Brats 2015 data. Zhao and Jia [53] also used a patch-wise CNN architecture using triplanar (axial, sagittal, coronal) 2D slices to segment brain tumors. They have obtained comparable results to state-of-art machine learning algorithms on Brats 2013 data. Kamnitsas et al. [21] presented a 3D dense-inference patch-wise and multi-scale CNN architecture that uses 3D (3 × 3 × 3 pixels) convolutional kernels and two pathway learning similar to [26]. They also used a 3D fully connected conditional random field to effectively remove false positives, which is an important post-processing step that was not described in previous studies. They reported the top ranking performance on Brats 2015. Dvorak et al. [54] presented a 2D patch-wise CNN approach that mapped input patches to n groups of structured local predictions that took into account the labels of the neighboring pixels. They reported results on Brats 2014 data that were comparable to those of state-of-art approaches. Most of these studies have also been presented in last two MICCAI conference as part of the BRATS challenge. We refer the reader to BRATS proceedings 2015–2016 [57] for further details such as performance comparison and ranking.

CNN-based deep learning architectures have also been used for segmentation of stroke and MS lesions, detection of cerebral microbleeds, and prediction of therapy response. Brosch et al. [31] presented a 3D semantic-wise CNN to segment MS lesions from MRI. They evaluated their method on two publicly available datasets, MICCAI 2008 and ISBI 2015 challenges, and compared their method to freely available and widely used segmentation methods. They reported performance comparable to the state of the art methods and superior to the publicly available MS segmentation methods. Dou et al. [32] presented a cascaded framework that included 3D semantic-wise CNN and a 3D patch-wise CNN to detect cerebral microbleeds (CM) from MRI. They reported their method outperformed previous studies with low level descriptors and provided a high sensitivity of 93.2% for detecting CM. Maier et al. [55] presented a comparison study that evaluated and compared nine classification methods (e.g., naive Bayes, random forest, and CNN) for ischemic stroke lesion segmentation. Their results showed that cascaded CNN and random decision forest approaches outperforms all other methods. Akkus et al. [29] presented prediction of 1p19q chromosomal co-deletion, which is associated with positive response to treatment in low grade gliomas from MRI using a 2D patch-wise and multi-scale CNN. The performance of their CNN approach on an unseen test set was 93.3% (sensitivity) and 82.22% (specificity) for detection of 1p19q status from MRI.

## Discussion

The recent advances reported in literature indicate significant potential for deep learning techniques in the field of quantitative brain MR image analysis. Even though deep learning approaches have been applied to brain MRI only recently, they tend to outperform previous state of the art classical machine learning algorithms and are becoming more mature. Brain image analysis has been a great challenge to computer-aided techniques due to complex brain anatomy and variability of its appearance, non-standardized MR scales due to variability in imaging protocols, image acquisition imperfection, and presence of pathology. Therefore, there is a need for more generic techniques such as deep learning that would handle these variabilities.

Despite a significant breakthrough, the potential of deep learning is limited because the medical imaging datasets are relatively small, and this limits the ability of the methods to manifest their full power, compared to what they have demonstrated on large-scale datasets (e.g., millions of images) such as ImageNet. While some authors report that their supervised frameworks require only one training sample [28], most researchers report that their results were consistently improving with an increase in size of training datasets [58, 59]. There is high demand for large-scale datasets for effective application of deep learning methods. Alternatively, the size of the dataset can be effectively increased by applying random transformations to the original data such as flipping, rotation, translation, and deformation. This is commonly used in machine learning and known as data augmentation. Data augmentation helps increase the size of training examples and reduce overfitting by introducing random variations to the original data. Multiple studies have reported the data augmentation to be very useful in their studies [9, 22, 29].

Several steps are crucial to improve the learning with deep learning approaches, including data preprocessing, data post-processing, network weight initialization, and strategies to prevent overfitting. Image preprocessing plays a key role in learning. Multiple preprocessing steps have been applied in current studies to improve learning process, as presented in Sections 2.5 and 2.6. For example, it is important to have intensities of input brain MR images in a reference scale and normalized for each modality. This avoids suppression of true patterns of structures by any modality and intensity differences in the output of the model. Post-processing of the output of model is also an important step to refine the segmentation results. The goal of any learning method is to have a perfect classification, but there are always regions in images that overlap between classes, known as partial volume effect, which unavoidably leads to false positives or negatives. These regions require additional processing for accurate quantification. Another important step is proper network parameter initialization in the neural network to maintain the gradient flow through network and to achieve convergence. Otherwise, the activations and gradient flow can vanish and result in no convergence and learning. Random weight initialization has been used in most of the current studies. Lastly, preventing overfitting is critical to learn the true information in images, and avoiding overfitting to specific training examples provided. Deep networks are particularly susceptible to overfitting because several thousands or millions of parameters are used in the networks and limited training data is available. Several strategies have been used to prevent overfitting such as data augmentation that introduces random variations to input data [9, 22, 29], using dropout that randomly removes nodes from network during training [22, 32, 54], and L1/L2 regularization that introduces weight penalties [26]. In current deep learning architectures, one or more of these strategies are used to prevent overfitting.

Semantic-wise architectures take inputs of any size and produce a classification map while patch-wise CNN architectures take fixed-sized inputs and produce non-spatial outputs. Therefore, semantic-wise architectures produce results for each pixel/voxel of an image much faster than patch-wise architectures. As presented in [60], it takes 22 ms to produce 10 × 10 grid of output from 500 × 500 input image for semantic-wise FCN while it takes 1.2 ms for patch-wise AlexNet [9] to infer a single value classification output of a 227 × 227 image, which is more than five times improvement in computation speed (22 vs. 120 ms). On the other hand, random sampling of patches over a dataset potentially results in faster convergence (LeCun et al. 1998) compared to full image training in semantic-wise architectures. Semantic-wise architectures also are more susceptible to class imbalance but this can be solved by weighting the classes in the loss function [31]. Cascaded architectures such as a patch-wise architecture following a semantic architecture as used in [32] would resolve the issues raised by each approach and refine the output results.

Developing a generic deep learning approach that will work on datasets from different machines and institutions is challenging due to limited training and ground truth data, variations and image acquisition protocols, imperfections of each MRI scanner, and variations in appearance of healthy and pathological brain tissue. So far, currently available methods were randomly initialized and trained on a limited data. To improve the generalization of deep learning architectures, one can adapt a well performing deep learning network trained on a large dataset and fine-tune that network on a smaller dataset specific to the problem, which is called transfer learning. It has been shown that transferring the weights (network parameters) from a pre-trained generic network to train on a specific dataset is better than random weight initialization of the network [61]. The usefulness and success of transfer learning depends on similarity between datasets. For instance, using pre-trained models from ImageNet, which is trained on a large RGB image database, might not perform well on medical images without further training. Shin et al. [62] reported that they obtained best performance with transfer learning from pre-trained model on ImageNet dataset and fine-tuning on lymph node and interstitial lung disease rather than training from scratch. On the other hand, the nature of the ImageNet dataset is much different than medical image dataset and therefore transfer learning from ImageNet might not the best choice for medical images as shown in [63].

## Summary

Despite the significant impact of deep learning techniques in quantitative brain MRI, it is still challenging to have a generic method that will be robust to all variations in brain MR images from different institutions and MRI scanners. The performance of the deep learning methods depends highly on several key steps such as preprocessing, initialization, and post-processing. Also, training datasets are relatively small compared to large-scale ImageNet dataset (e.g., millions of images) to achieve generalization across datasets. Moreover, current deep learning architectures are based on supervised learning and require generation of manual ground truth labels, which is tedious work on a large-scale data. Therefore, deep learning models that are highly robust to variations in brain MRI or have unsupervised learning capability with less requirement on ground truth labels are needed. In addition, data augmentation approaches that realistically mimic variations in brain MRI data could alleviate the need of large amount of data. Transfer learning could be used to share well-performing deep learning models, which are trained on normal and pathological brain MRI data, among brain imaging research community and improve the generalization ability of these models across datasets with less effort than learning from scratch.
